# Supplementary material for: COVID-19 Causes Ferroptosis and Oxidative Stress in Human Endothelial Cells
Source: Antioxidants (Basel). 2023 Jan 31;12(2):326. doi: 10.3390/antiox12020326 (PMC9952002; doi:10.3390/antiox12020326)
Supplement: Supplementary file 1 [file antioxidants-12-00326-s001.zip › antioxidants-2102478-supplementary.pdf]

Supplementary Material

COVID-19 causes ferroptosis and oxidative stress in human endothelial cells

Stanislovas S. Jankauskas, Urna Kansakar, Celestino Sardu, Fahimeh Varzideh, Roberta Avvisato, Xujun Wang, Alessandro Matarese, Raffaele Marfella, Marcello Ziosi, Jessica Gambardella, and Gaetano Santulli

	Survivors	Non-Survivors
**Number of patients**	22	20
**Age (years)**	62±8.4	63±14
**Sex: Male/Female (%)**	74/26	72/28
**Time to death after admission and blood sampling (days)**	n/a	17.4±16.7

Table S1. Main characteristics of COVID-19 patients from whom the serum was obtained. Data are mean ± SD or percentage. *: $p<0.01$.

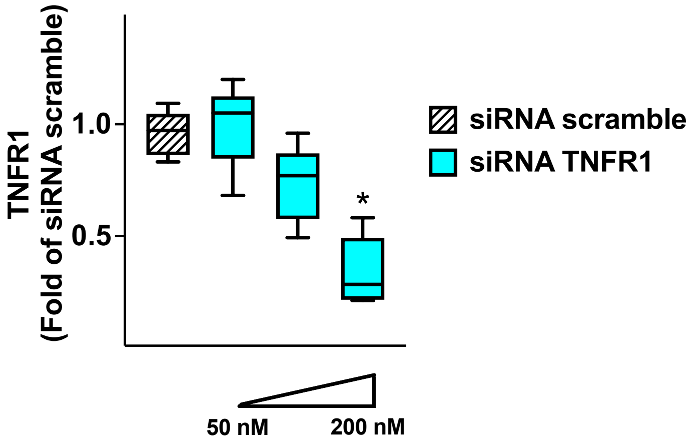

Figure S1

Successful knock-down of TNFR1 in human endothelial cells. HUVECs were treated for 48 h with siRNA scramble or siRNA TNFR1 to test TNFR1 silencing in dose-response assays (data were normalized for the housekeeping gene GAPDH; *:$p<0.01$ *vs* siRNA scramble). Three different doses of TNFR1 siRNA were tested: 50, 100, and 200 nM. The experiments were performed at least in triplicate; the box-and-whiskers graph shows the median and the 5th-95th percentiles. Sequences of oligonucleotide primers are listed in Table 1.
